# Editorial: Insights in ethnopharmacology: 2022

**DOI:** 10.3389/fphar.2023.1264063

**Published:** 2023-08-29

**Authors:** Cheorl-Ho Kim, Michael Heinrich, Hung-Rong Yen, Javier Echeverria, Aiping Lu

**Affiliations:** ^1^ Department of Biological Sciences, College of Science, Sungkyunkwan University, Suwon, Republic of Korea; ^2^ School of Pharmacy, University College London, London, United Kingdom; ^3^ China Medical University (Taiwan), Taichung, Taiwan; ^4^ Departamento de Ciencias Del Ambiente, Facultad de Química y Biología, Universidad de Santiago de Chile, Santiago, Chile; ^5^ Hong Kong Baptist University, Kowloon, Hong Kong SAR, China

**Keywords:** insights in ethnopharmacology: 2022, ethnomedicinal drugs, preventive strategy, therapeutic efficacy, phytochemicals, genomic sequencing technology, combinatory pharmacology

In the third decade of the 21st century, scientists continue to achieve tremendous accomplishments in the field of ethnopharmacology/medicinal plant research (Kim et al.). The Frontiers Research Topic *“Insights in ethnopharmacology: 2022”* brings together recent advances in natural product research and ethnopharmacology, detailing the progress and innovation in recent decades. Within Frontiers in Pharmacology, the focus is very much on developing scientific understanding of all drugs, including those derived from natural sources.

In the review “Horizon scan of DNA-based methods for quality control and monitoring of herbal preparations” (Raclariu-Manolica et al.), recent developments in emerging DNA-based technologies are discussed, focusing on the opportunities they provide for authenticating herbal substances. The authors developed a method for authentication and monitoring, a promising tool for simultaneous identification of medicinal plant resources. The review provides a vision of the recent achievements and advances in emerging DNA-based technologies, assessing them in the context of ethnopharmacology and medicinal plant research. Of course, authentication is the foundation for any evaluation of the pharmacologic effects, safety, and clinical efficacy of specific preparations, plant-derived dietary supplements, and other preparations in the form of crude extracts and polyherbal preparations (Heinrich et al.). There remains a lack of consensus on the description and characterization of pharmacologically and biologically active extracts and metabolites, often at a very fundamental level. Jordan *et al.* (2023), for example, raise concerns about the authenticity of some “botanicals” as well as unexpected contamination and profit-based artificialization. Simultaneously, scientific evidence is used to achieve general acceptance and agreement, mostly via an authorization by the FDA of poorly characterized medicinal or dietary products. Thus, analytical technologies such as DNA barcoding need to be incorporated into routine quality assurance not only of licensed herbal medicines, but also supplements and “botanicals.” Academia, manufacturing industries, and regulatory bodies must cooperate with each other to define and make strategic overviews and policies for the authentication and identification of plants, botanical extracts, their formulation, and applicable preparations to ensure medicinal drug QC. DNA/RNA-based technologies have made important contributions to ethnopharmacology, and their further development is a core task for the future.

*Cyclopia subternata* Vogel extracts contain downregulators specific for the selective estrogen receptor (ER) subtype specifically expressed in ER-positive breast cancer (BC) cells by comparison with BC endocrine therapies and selective ER antagonists and agonists (Olayoku et al.; Visser et al., 2023). Medicines acting on BC cells with lower toxicities, less resistance induction, and fewer side effects, but as effective as current therapies from the indigenous South African fynbos plant of the Cyclopia species, have been suggested. The plant extracts contain phenolic compounds with chemopreventive activities as well as with phytoestrogenic antigenic potentials, which are effective against BC development, growth, proliferation, and progression (Ondo et al., 2012). ER-α activates BC cell growth, whereas ER-β reverses and suppresses the ER-α–elicited growth activity. Considering that BCs proliferate via ER-α in ER + breast cancers, endocrine therapies using aromatase inhibitors, selective ER modulators (SERMs), and selective ER down-regulators (SERDs) mainly target ER + breast cancers. Although their effectiveness is well-known, their endocrine therapy demerits are mainly due to their severe side effects and resistance. The authors examined *Cyclopia* extracts (SM6Met, CoT, and P104) for their capacity to downregulate the ER-α and ER-β expressions associated with BC prognosis and treatment. *C. subternata* extracts, named SM6Met and CoT, decreased the ERα but increased the ERβ protein expression level. Thus, a reduced ERα:ERβ ratio is essential for standard of care BC endocrine therapies, such as the known SERD fulvestrant and SERM 4-hydroxytamoxifen. The downregulated ERα and ERβ levels caused by Cyclopia extracts have been understood at the mRNA and protein levels as well as at the proteasomal degradation level, specifically modulating the ER subtype levels to inhibit BC growth as potential BC therapeutics.

A review on the functional role of phosphodiesterase-1 (PDE1) focuses on natural product and plant-derived inhibitory agents in Alzheimer’s Disease (AD), with an emphasis on the role of PDE1 inhibitors. Ahmad et al. systematically explore the clinical importance of, research, and clinical trials on the prevention and therapy of AD. PDE1 and its isoforms show characteristic patters in terms of splicing variants, location, distribution, and function. PDE1 inhibitory agents could become an innovative therapeutic approach for the treatment and prevention of AD. The current absence of available selective preventive and therapeutic medicines requires approaches to explore the efficacy, beneficial effects, safety profile, and profits of natural plant-derived products. PDE1 inhibition has been suggested to be an AD-therapeutic option by other scholars such as Shekarian *et al.* (2023). Ahmad et al. comprehensively reviewed the biological and pharmacological properties of PDE1, focusing on its role in AD and its importance as an AD-targeted therapy in drug creation and discovery from natural plant-derived products. Natural products and vinpocetine have been compared as PDE1 inhibitors, providing current information and future perspectives. The review greatly enhances trials in the clinic and scientific approaches focusing on the prevention and therapeutic treatment of AD.

Mavungu et al. focus on an emerging area of ethnopharmacological research—the use of herbal medicines in managing livestock diseases and their prevention. The authors document and analyze the use of medicinal plants in three territories of the Haut-Katanga province, Congo, and assess the current ethnopharmacological evidence. Several species are identified, which should have priority for future research and development. Similar efforts are now under way in many African, Asian, and other countries (e.g., Rahman et al., 2023).

In conclusion, this small Research Topic shows the diversity of approaches in the field and the opportunities arising from medicinal plant research. Investigations on natural products, herbal medicines, and genomic DNA sequence-based authentication of plant ingredient–based prediction, as well as action mechanisms and pharmacology predisposing targets in the context of “ethnopharmacology in human diseases: therapeutic and preventive actions,” show the current profile of ethnopharmacology, emerging therapeutic trends, and research needs. The studies open new vistas for pragmatic solutions in an ethnopharmacological context. Moreover, the articles provide insights into future preventive and treatment strategies using classical and advanced therapeutic strategies.
